# Genetic reconstruction of a bullfrog invasion to elucidate vectors of introduction and secondary spread

**DOI:** 10.1002/ece3.2278

**Published:** 2016-06-28

**Authors:** Pauline L. Kamath, Adam J. Sepulveda, Megan Layhee

**Affiliations:** ^1^ Northern Rocky Mountain Science CenterU.S. Geological Survey2327 University Way, Suite 2BozemanMontana59715

**Keywords:** Biological invasion, bullfrogs, cytochrome *b*, dispersal, invasive species, *Lithobates catesbeianus*, phylogeography, population genetics

## Abstract

Reconstructing historical colonization pathways of an invasive species is critical for uncovering factors that determine invasion success and for designing management strategies. The American bullfrog (*Lithobates catesbeianus*) is endemic to eastern North America, but now has a global distribution and is considered to be one of the worst invaders in the world. In Montana, several introduced populations have been reported, but little is known of their sources and vectors of introduction and secondary spread. We evaluated the genetic composition of introduced populations at local (Yellowstone River floodplain) and regional (Montana and Wyoming) scales in contrast to native range populations. Our objectives were to (1) estimate the number of introductions, (2) identify probable native sources, (3) evaluate genetic variation relative to sources, and (4) characterize properties of local‐ and regional‐scale spread. We sequenced 937 bp of the mitochondrial cytochrome *b* locus in 395 tadpoles collected along 100 km of the Yellowstone River, from three additional sites in MT and a proximate site in WY. Pairwise Φ_ST_ revealed high divergence among nonnative populations, suggesting at least four independent introductions into MT from diverse sources. Three cyt *b* haplotypes were identical to native haplotypes distributed across the Midwest and Great Lakes regions, and AMOVA confirmed the western native region as a likely source. While haplotype (*H*_d_ = 0.69) and nucleotide diversity (*π* = 0.005) were low in introduced bullfrogs, the levels of diversity did not differ significantly from source populations. In the Yellowstone, two identified haplotypes implied few introduction vectors and a significant relationship between genetic and river distance was found. Evidence for multiple invasions and lack of subsequent regional spread emphasizes the importance of enforcing legislation prohibiting bullfrog importation and the need for continuing public education to prevent transport of bullfrogs in MT. More broadly, this study demonstrates how genetic approaches can reveal key properties of a biological invasion to inform management strategies.

## Introduction

Retracing historical colonization pathways provides fundamental insights into the origin and genetic composition of species invasions and the factors determining invasion success (Dlugosch and Parker 2008; Estoup and Guillemaud 2010). Phylogeographic and population genetic approaches have proven to be valuable for revealing important information such as the number of sources, introductions and founding individuals, the genetic composition of both source and invasive populations, and the dynamics of secondary spread. Genetic variability, in particular, is expected to be a key determinant of invasion success, enabling adaptive potential and persistence in new environments (Kolbe et al. 2004; Lavergne and Molofsky 2007); however, invasion events often involve a low number of founders and are subject to genetic bottlenecks (e.g., Ficetola et al. 2008). Paradoxically, there are numerous examples of invasion successes despite low levels of population genetic variation (reviewed in Novak and Mack 2005; Wares et al. 2005). Empirical studies have also shown that not all invasions are accompanied by reduced levels of genetic diversity (reviewed in Wares et al. 2005; Roman and Darling 2007). Hence, the role of genetic bottlenecks, multiple introductions, and gene flow among introduced populations in colonization success remains unclear.

Understanding the history of the invasion process also has practical implications for informing the design of prevention and control strategies (Estoup and Guillemaud 2010). If the geographic origin and the vectors responsible for the introduction of an invader can be identified, then prevention and monitoring measures targeting specific source areas and pathways can be implemented. For example, knowledge that trailered recreational boats are a primary vector for the inland spread of invasive bivalves (*Dreissena* spp.) has prompted mandatory boat inspection stations at many water bodies and at jurisdictional borders (Johnson et al. 2001; Rothlisberger et al. 2010; Kelly et al. 2013). The environmental and economic impacts of invasive species are costly (nearly $120 billion per year in the United States alone; Pimentel et al. 2005) and often irreversible, so prohibiting the initial entry of invasive species into a new range has become the cornerstone of most invasive species management programs. As such, many regions have developed biosecurity infrastructures and regulations aimed at preventing the initial entry of invasive species.

While prevention of initial introductions has been the focus of many programs, less attention has been given to the issue of secondary spread of invasive species once they have already established (Kowarik 2003; Vander Zander and Olden 2008; Paini et al. 2010). Intentional or accidental secondary releases within the new range can be made over long periods subsequent to the initial introduction and can result in high rates of establishment, population growth, range expansion and, concomitantly, large environmental and economic impacts (Kowarik 2003; Rothlisberger et al. 2010). For invasive species that are already established, effective allocation of management effort toward prevention of new initial introductions versus prevention of secondary releases is critical given the rising number of potential and established invaders, the major environmental and economic damages these species can cause, and the reality of limited resources.

**Figure 1:**
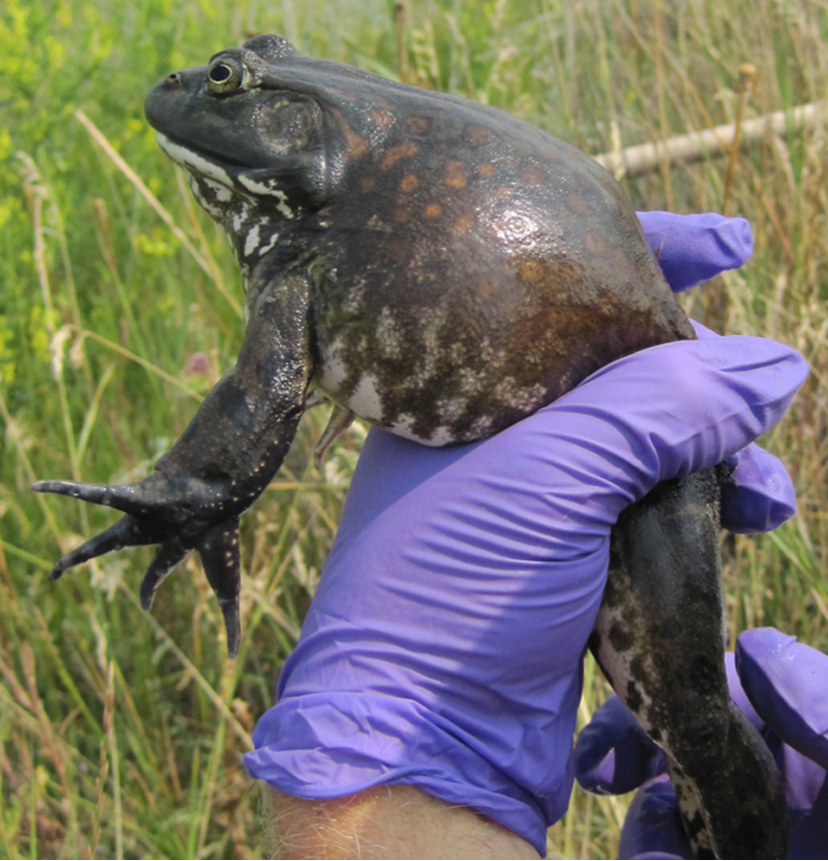
An adult American bullfrog (*Lithobates catesbeianus*) captured in Grand Teton National Park in July 2015. Bullfrogs were first documented in this park in a geothermally influenced pool in the 1950s and have since spread 4 km downstream. Photograph credit: Adam Sepulveda, U.S. Geological Survey.

American bullfrogs (*Lithobates catesbeianus* [Shaw, 1802]; hereafter bullfrog) have been introduced across the globe and are listed as one of the 100 worst invasive species (Lowe et al. 2000; Fig. 1). Therefore, bullfrogs have been an important focal study species for examining the properties of invasion and spread (Ficetola et al. 2007b). Bullfrogs are native to eastern North America (Bury and Whelan 1984), but now occur in nearly all lower 48 states and in over 40 countries. Their large size, high mobility, generalist eating habits, high fecundity, and function as a disease vector to other amphibians make the bullfrog an extremely successful invader and a major threat to biodiversity (Adams and Pearl 2007; Miaud et al. 2016). For these reasons, evaluating sources, genetic properties and historical invasion pathways are key to the development of effective policy and management strategies needed to minimize their ecological impacts.

Previous studies using genetic approaches have found that the history of bullfrog introductions and secondary spread varies greatly among regions. Source populations for European bullfrogs were from the western and eastern portions of the native range (Ficetola et al. 2008), while source populations for Oregon's Willamette Valley were from the central portions of the native range (Funk et al. 2011). In contrast, the source of China's bullfrog population was a nonnative population in Cuba (Bai et al. 2012). Both European and Chinese bullfrogs had low haplotype diversity among and within sampled populations and haplotypes were similar among populations; this suggests that bullfrogs were translocated after their introduction and that bullfrogs can be successful colonizers in spite of low genetic diversity. For these regions, management to prevent secondary spread of bullfrogs may be as important as management to prevent initial introductions.

Bullfrogs were introduced to western Montana prior to the 1960s for human consumption and are now widespread in the Flathead and Clark Fork drainages (Black 1969; Maxwell 2000). More recently, bullfrog populations were documented in the Yellowstone and Tongue River drainages of eastern Montana in 1999 and 2004, respectively (Sepulveda et al. 2015). It is not known whether these recent populations were translocated from western Montana or came from outside the state. However, this knowledge is urgent for prioritizing the allocation of resources to prevent future introductions and spread, particularly given that Montana waters exhibit high suitability for bullfrogs (Ficetola et al. 2007b).

Here, we used phylogeographic and population genetic approaches to reconstruct the invasion history of bullfrogs in Montana and characterize their genetic properties for the purposes of (1) identifying the number of introduction events, sources, and genetic diversity of a successful invasion and (2) providing guidance about how to allocate limited management resources between prevention of new bullfrog introductions and control of secondary spread from established invasive populations in the region. In Montana, state border inspection stations and regulations that identify species as prohibited or regulated are the primary tools for preventing the initial entry of invasive species, whereas management approaches to limit the secondary spread of bullfrogs within the state are uncommon.

If the genetic data indicate support for within‐state translocation, then control of established populations and outreach education should be prioritized. In contrast, if data suggest that bullfrog populations in Montana are genetically distinct from one another, then efforts to stop bullfrog importation into the state should continue to be prioritized and bolstered. In order to distinguish between the two scenarios, we specifically used mitochondrial sequence data to (1) estimate the number of introductions, (2) identify probable native population sources, (3) determine whether genetic variation is reduced relative to sources, and (4) characterize the properties of local‐ and regional‐scale secondary spread.

## Materials and Methods

### Sample collection and DNA extraction

**Figure 2:**
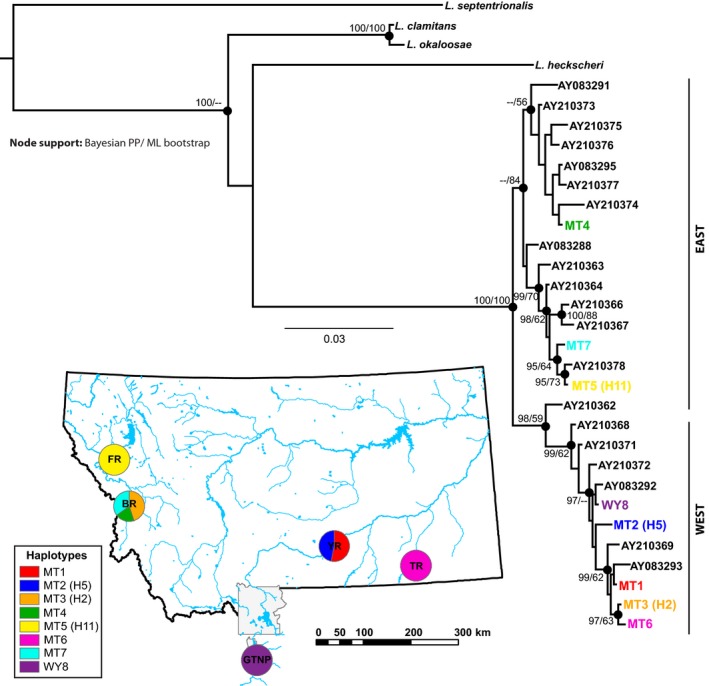
Bayesian consensus *Lithobates catesbeianus* mtDNA cytochrome *b* phylogeny. Node support is reported in terms of Bayesian posterior probabilities (PP) and maximum‐likelihood (ML) bootstrap values (only PP > 95% and ML bootstrap support values >50% are shown). Scale bar indicates genetic distance in units of nucleotide substitutions per site. The tree was rooted with *Lithobates septentrionalis* and included outgroup sequences from *Lithobates clamitans*,* Lithobates okaloosae*, and *Lithobates heckscheri* (GenBank accession nos.: AY083273, AY083281, AY083286, AY083299). Previously reported native range bullfrog sequences are identified by their GenBank accession number. Haplotypes identified in this study are represented in corresponding colors on the tree and map, with map showing haplotype composition at each sampling site. Identity to haplotypes from Austin et al. (2004) (H2, H5, H11) is shown in parentheses. FR, Flathead River; BR, Bitterroot River; YR, Yellowstone River; TR, Tongue River; GTNP, Grand Teton National Park (Kelly Warm Springs).

**Figure 3:**
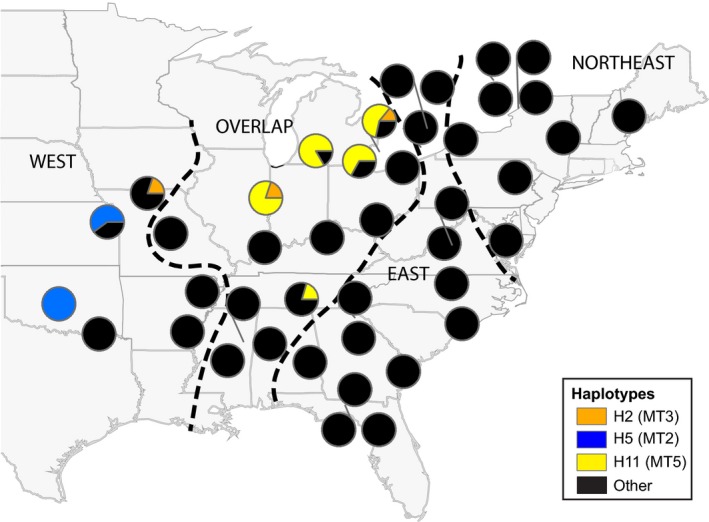
*Lithobates catesbeianus* native range sources from the eastern United States. Native range distributions of mtDNA cyt *b* haplotypes identical to those found in Montana (in parentheses) are shown with colors corresponding to Figure 1: orange = haplotype 2 (H2); blue = haplotype 5 (H5); yellow = haplotype 11 (H11). Other haplotypes are shown in black. Native range haplotype data and genetic regions (west, overlap, east, northeast) as defined by nested clade analysis are derived from Austin et al. (2004).

We obtained a total of 368 bullfrog tadpole tissue samples between 2013 and 2014 from four river floodplains in Montana: Bitterroot River (*n *=* *40); Flathead River (*n *=* *5); Tongue River (*n *=* *15); and Yellowstone River (YR, *n *=* *308; Table 1; Fig. 2). These populations are representative of the currently established bullfrog populations in Montana. We sampled the YR more intensively, collecting tadpoles at 33 sites (*n *=* *1–30 samples/site; Table 2, Fig. 3) that spanned 100 river km, as part of a more detailed investigation of bullfrog spread at a local scale (see Sepulveda et al. 2015). We also obtained 27 tadpole tissue samples from Kelly Warm Springs in Grand Teton National Park (GTNP) within Wyoming. Because this introduced population is proximate to Montana and has been established since the 1950s, it represents a potential source from outside the state. We extracted total genomic DNA from bullfrog tissue using the DNeasy Tissue Kit (Qiagen Inc., Valencia, CA), following the manufacturer's instructions.

**Table 1:** Population genetic diversity of the cytochrome *b* locus within introduced *Lithobates catesbeianus* populations

Population	*n*	*S*	*A*	*H*_d_ (SD)	*π* (SD)
Bitterroot River	40	21	3	0.651 (0.034)	0.011 (0.006)
Flathead River	5	0	1	0.000 (0.000)	0.000 (0.000)
Tongue River	15	0	1	0.000 (0.000)	0.000 (0.000)
Yellowstone River	308	5	2	0.500 (0.004)	0.003 (0.002)
Grand Teton NP	27	0	1	0.000 (0.000)	0.000 (0.000)
All	395	26	8	0.687 (0.015)	0.005 (0.003)

Diversity indices reported in terms of number of polymorphic sites (*S*), allelic diversity (*A*), haplotype diversity (*H*_d_), and nucleotide diversity (*π*). Population sample size (*n*) and standard deviations for *H*_d_ and *π* are shown.

**Table 2:** Pairwise Φ_ST_ (above diagonal) and associated *P*‐values (below diagonal) between introduced *Lithobates catesbeianus* populations in Montana and Grand Teton National Park, Wyoming

	Bitterroot	Flathead	Tongue	Yellowstone	Grand Teton
Bitterroot	–	0.33	0.44	0.60	0.43
Flathead	0.0059	–	1.00	0.87	1.00
Tongue	<0.0001	0.0002	–	0.56	1.00
Yellowstone	<0.0001	<0.0001	<0.0001	–	0.34
Grand Teton	<0.0001	<0.0001	<0.0001	<0.0001	–

All Φ_ST_ values were significant after applying a sequential Bonferroni correction for multiple tests.

### Cytochrome *b* amplification and sequencing

We sequenced the mitochondrial DNA (mtDNA) cytochrome *b* (cyt *b*) locus to reconstruct pathways of bullfrog introductions and secondary spread within Montana. We performed polymerase chain reaction (PCR) to amplify a 1047‐base pair (bp) segment of the cyt *b* gene using the MVZ15‐L (Moritz et al. 1992) and cyt‐bAR‐H (Goebel et al. 1999) primers. The targeted region includes the 408‐bp segment used by Austin et al. (2004) to characterize the phylogeographic diversity of bullfrogs across their native range. Other studies have employed this marker for investigating the species' invasion history (Ficetola et al. 2008; Funk et al. 2011; Bai et al. 2012). PCRs were carried out in a 15 μL total reaction volume which consisted of approximately 25 ng template DNA, 2 mmol/L MgCl_2_, 200 μmol/L deoxynucleotide triphosphates, 5 μL of 5× GoTaq polymerase buffer (Promega, Madison, WI, USA), 0.6 U of GoTaq DNA polymerase (Promega, Madison, WI, USA), 1 μmol/L of each primer, and distilled sterile H_2_O to volume. The following thermocycling conditions were used: initial denaturation at 94°C for 6 min; 35 cycles of 94°C for 20 s, 52°C for 30 s, and 72°C for 30 s; and final extension at 72°C for 5 min. We included a negative control in all PCR runs. Amplified PCR products were purified using Exo‐SAP‐IT (Affymetrix, Cleveland, OH, USA) and cycle‐sequenced in both directions using forward and reverse primers. We submitted purified products to GENEWIZ (http://www.genewiz.com/) for custom sequencing.

### Population genetic diversity

We estimated measures of population genetic diversity and differentiation (*F*‐statistics) in Arlequin 3.5 (Excoffier and Lischer 2010). Genetic diversity indices included the number of polymorphic sites (*S*), allelic diversity (*A*), haplotype diversity (*H*_d_; Nei 1987), and nucleotide diversity (*π*; Nei 1987). We further estimated both *H*_d_ and *π* of populations within potential source regions of the native range using data from (Austin et al. 2004).

### Phylogenetic analysis

We edited and aligned cyt *b* sequences using Geneious ver. 6 (http://www.geneious.com; Kearse et al. 2012). The final sequence alignment was trimmed to a length of 923 bp. We reconstructed phylogenetic relationships among cyt *b* haplotype sequences identified in introduced bullfrog populations of Montana and GTNP, and placed these phylogenetic relationships in a broader context with 23 sequences previously identified in bullfrogs collected from North American native range sites (see Table S1 in Supporting Information; Austin et al. 2003, 2004). We reduced the data alignment to unique haplotypes and used sequences from *Lithobates clamitans*,* Lithobates heckscheri, Lithobates okaloosae*, and *Lithobates septentrionalis* (see Table S1) as outgroups. The best‐fit model of nucleotide substitution was identified using Akaike information criteria (Akaike 1974) as implemented in jModelTest ver. 2.1 (Posada 2008).

Phylogenetic reconstructions were estimated using both Bayesian inference and a maximum‐likelihood (ML)‐based approach. Bayesian inference involved a four‐chain Metropolis‐coupled Markov chain Monte Carlo analysis as implemented in MrBayes ver. 3.2 (Ronquist et al. 2012). Two independent analyses were run in parallel for 5 million generations with the heating parameter set to 0.1. Parameters and trees were sampled every 100 generations. The consensus tree was estimated using combined results from the two runs, after discarding the first 25% of trees as “burn‐in.” We evaluated convergence and stationarity of Bayesian runs by ensuring that the standard deviations of split frequencies approached 0 (<0.005) and the potential scale reduction factor was 1 for all parameters. We further confirmed relationships among bullfrog haplotypes using a ML approach in RAxML ver. 8 (Stamatakis 2014). We conducted 100 runs, and ML branch support was evaluated by 10,000 bootstrap replicates. Well‐supported nodes were identified as having a Bayesian posterior probability (PP) >95% or ML bootstrap support >50%.

### Evaluating bullfrog introduction history

The minimum number of introductions into the region was determined by estimating Φ_ST_ between pairs of introduced populations, with significance evaluated by performing 10,000 permutations. We applied a sequential Bonferroni correction (Holm 1979) to adjust the critical *P*‐value for multiple (*n *=* *10) pairwise tests. A significant pairwise Φ_ST_ reflected two populations that were genetically distinct and, following Funk et al. (2011), were considered to reflect independent introductions. In contrast, chains of nonsignificant tests may be indicative of samples derived from the same genetic population (Waples and Gaggiotti 2006). Because lack of differentiation could be caused by a single introduction or multiple introductions from the same source population, this analysis was only capable of determining the minimum number of introductions. Also, lack of differentiation between populations does not necessarily exclude the possibility of connectivity among introduced populations given that introductions are typically established by a small number of founders.

We evaluated possible native range source populations for bullfrog invasion in Montana and GTNP based on two lines of evidence. First, we examined the geographic distribution of identical haplotypes in the native range, as reported in Austin et al. (2004), based on both full‐length and truncated (408 bp) data. Second, we applied an analysis of molecular variance (AMOVA; Excoffier et al. 1992) to evaluate differentiation and the amount of variance that could be attributed to differences between introduced and native range populations (May et al. 2006). Native range data (see Table S1) were grouped into four regions (west, overlap, east, and northeast; Fig. 3), which were previously defined by Austin et al. (2004) using a nested clade analysis. We used pairwise differences to calculate the distance matrix, and significance was determined through 10,000 permutations.

We assessed whether there was a significant difference in diversity indices among introduced and identified native range source populations. Genetic diversity indices used in comparisons were haplotype and nucleotide diversity, and differences in median diversity among native and introduced regions were tested using Mann–Whitney *U*‐tests.

### Isolation by distance in the Yellowstone River

We constructed generalized linear models for each haplotype identified in the YR, with each haplotype proportion as the response variable and river distance (in km) from the most upstream site (3_11; see Table S2) as the explanatory variable. We specified a binomial error structure and weighted the data by site sample size. In addition, we performed a generalized additive model to examine the relationship between site pairwise *F*_ST_ and river distance in the mgcv package (Wood 2004). Variation in the fitted trend due to uncertainty in model parameter estimates was visualized by conducting 1000 simulations drawing from the posterior distribution of the parameters. We examined genetic isolation by distance by performing a Mantel's test (Mantel 1967) that tested the correlation between genetic and geographic distance matrices in the ecodist package (Goslee and Urban 2007). Pairwise genetic *F*_ST_ was calculated among all pairs of sampling sites in the YR using Arlequin ver 3.5 (Excoffier and Lischer 2010). Pairwise geographic river distances were extracted from ArcMap, after snapping sites located in the floodplain to the nearest perpendicular location on the river. The 95% confidence interval (CI) of the Mantel test statistic (Mantel's *R*) was determined by 1000 bootstrap replicates and significance assessed with 10,000 permutations. We constructed a Mantel correlogram to explicitly consider the spatial structure at different distance classes spaced at 5‐km intervals. All statistical analyses were conducted using R (R Development Core Team 2015).

## Results

### Indices of population genetic diversity

We identified eight cyt *b* haplotypes in introduced bullfrog populations of Montana and GTNP (MT1 to MT7, WY8; Fig. 2, Table S1, GenBank accession nos. KX34485–KX34492). All haplotypes were private within populations in the study region, each only found in a single location (Table S3). Three haplotypes were identical to those previously discovered in native range bullfrogs (MT2 = H5; MT3 = H2; MT5 = H11; Fig. 3). When truncating the cyt *b* sequence data to the same 408 bp used in Austin et al. (2004), we identified fewer (6) haplotypes in Montana and GTNP, with a larger geographic overlap of identical haplotypes extending to the Northeastern native range (Fig. S1). The Bitterroot population exhibited the highest genetic diversity (*A *=* *3, *H*_d_ = 0.651, *π *= 0.011; Table 1), whereas in the YR, we observed low diversity (*A *=* *2, *H*_d_ = 0.5, *π *= 0.003; Table 1). In three invasive populations (Flathead, Tongue, GTNP), no diversity was observed.

### Bullfrog cyt *b* phylogenetic tree

The best‐fit model of nucleotide substitution was HKY + Γ with the following likelihood parameters: ln *L *=* *−2571.06; transition/transversion ratio = 11.42; shape parameter = 0.244. We specified this model when reconstructing phylogenetic relationships. The cyt *b* haplotypes sampled from invasive populations in Montana and GTNP were interspersed within both eastern and western cyt *b* lineages from the native range (Fig. 2). However, the majority of haplotypes (MT1, MT2, MT3, MT6, WY8) were nested within the western lineage, whereas three haplotypes (MT4, MT5, MT7) were nested within the eastern lineage. We observed moderate‐to‐high support for monophyly of the western lineage (Bayesian PP = 98%, ML bootstrap = 59%). The eastern lineage was also monophyletic, but support was low to moderate (Bayesian PP = 42%, ML bootstrap = 84%). Clustering of MT3 and MT6 suggests these haplotypes may share a common source.

### Introduction history of bullfrogs into Montana

Population differentiation was high and significant, after sequential Bonferroni correction, for all population pairs (Φ_ST_ range: 0.33–1, *P*‐value range: <0.0001–0.0059; Table 2). These results suggest a minimum of four independent bullfrog invasions into Montana originating from outside the study region. The GTNP population was significantly differentiated from all Montana populations, indicating that GTNP is not a source, or vice versa, but instead represents a separate bullfrog invasion into the region.

The three native haplotypes that were identical to haplotypes found in this study (i.e., H2, H5, and H11 from Austin et al. (2004)) had a native range distribution across localities in both the west and overlap regions of the native range. These haplotypes were primarily clustered in the Midwest and Great Lakes regions of the United States (Fig. 3). AMOVA results revealed no among‐group variation between introduced bullfrogs and the west native region (*P *=* *0.72), confirming that the bullfrog invasion in Montana and GTNP most likely originated from this region (Table 3). Low, marginally significant among‐group variation was observed in comparison with the overlap region (31.4%, *P *=* *0.09). In contrast, there was significant among‐group variation between the introduced and the east (61.2%, *P *<* *0.001) or northeast (70.6%, *P *<* *0.001) native bullfrog regions.

**Table 3:** Analysis of molecular variance results showing the percentage of among‐group variation between introduced and native range bullfrog populations

Native region compared to introduced bullfrogs	Percentage of among‐group variation	*P*‐value
Northeast	70.6	<0.001
East	61.2	<0.001
Overlap	31.4	0.088
**West**	**0.0**	**0.723**

The most likely native region origin (shown in bold) was identified as having the lowest, nonsignificant among‐group variation when compared with introduced bullfrogs.

Genetic diversity of introduced populations within the introduced range of Montana and GTNP was very low (median *H*_d_ = 0.000, median *π *= 0.00), relative to the west (median *H = *0.650, median *π *= 0.003) and overlap (median *H*_d_ = 0.700, median *π *= 0.010) native range regions. However, Mann–Whitney *U*‐tests revealed that the levels of genetic diversity were not significantly different than west native source populations (*H*_d_: *P *=* *0.14, *π*:* P *=* *0.79, df = 9). In contrast, *H*_d_ was significantly lower in introduced versus potential overlap source populations (*P *=* *0.01, df = 16), whereas differences in *π* were only marginally significant (*P *=* *0.06, df = 16).

### Bullfrog genetic and spatial structure along the Yellowstone River

**Figure 4:**
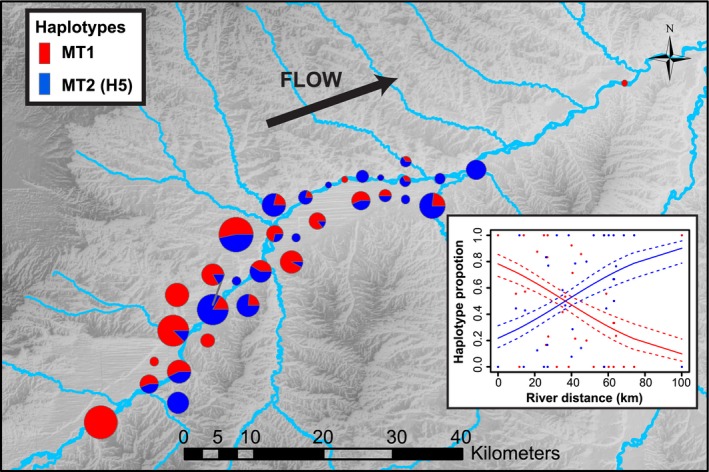
Spatial distribution of *Lithobates catesbeianus* mtDNA cyt *b* haplotypes along the Yellowstone River, MT. Pie charts on map show proportions of each haplotype sampled (MT1: red, MT2/H5: blue), with circle size relative to sample size (*n *=* *1–30). Inset shows generalized linear model prediction of haplotype proportion as a function of distance from most upstream sampling location. Dashed lines represent 95% confidence intervals.

**Figure 5:**
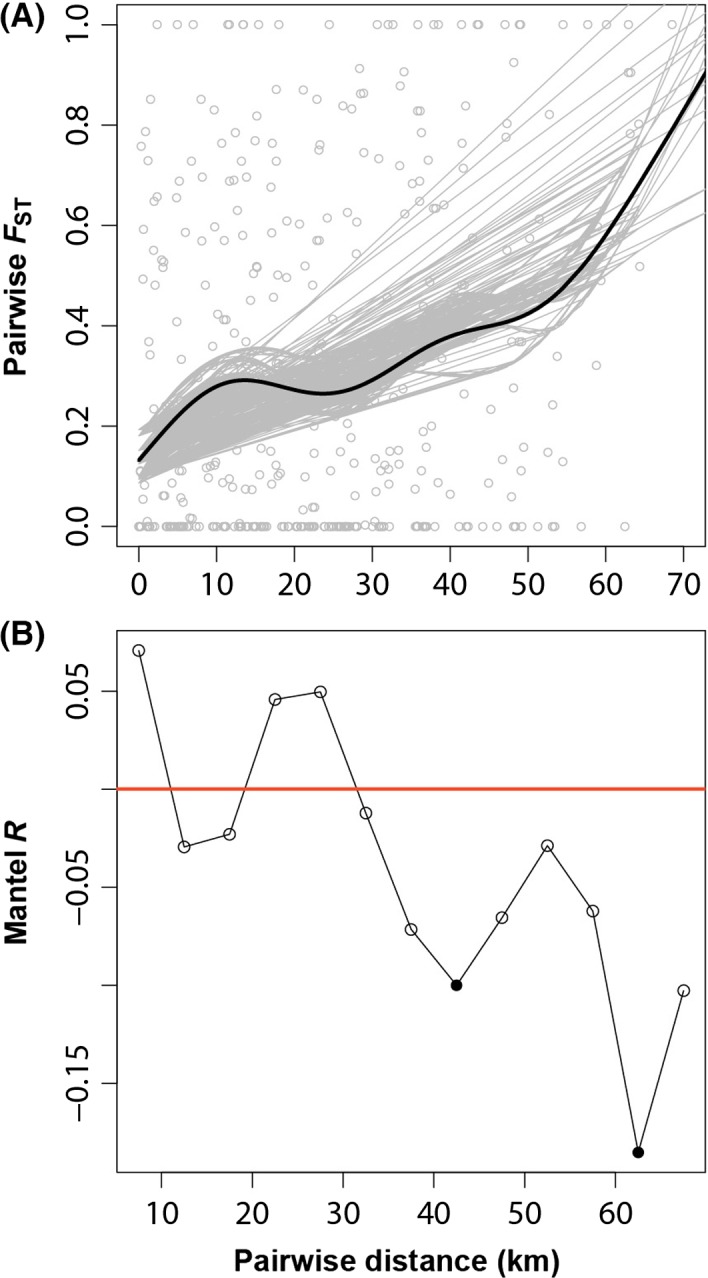
Relationship between genetic distance and river distance (A) and Mantel correlogram showing spatial structuring at different distance classes (B). Variation in the fitted trend of the generalized additive model (in A) is represented by 100 randomly selected subsets drawn from the posterior distribution of the parameters. Significance (in B) is represented by the closed circles.

In the YR, we found a significant relationship between haplotype (MT1, MT2) proportion and river distance (*P *<* *0.0001), with the proportion of haplotype MT1 decreasing and haplotype MT2 increasing with distance from the most upstream site (Fig. 4; Table S2). There was also a positive relationship between population pairwise *F*_ST_ and geographic river distance among sites along the YR (Fig. 5A). A Mantel's test further confirmed a significant relationship between genetic (i.e., *F*_ST_) and geographic distance (Mantel's *R *=* *0.28, 95% CI: 0.17–0.37, *P *=* *0.0002). Inspection of the Mantel correlogram revealed a roughly linear pattern, with an inverse relationship between Mantel's R and distance between sites (Fig. 5B). Sites in close proximity (i.e., the first distance class, 5–10 km) tended to be more genetically similar than sites further away. At greater distances (>30 km), the degree of genetic dissimilarity between pairs of sites increased. However, Mantel's *R* values were only significant at the 40–45 and 60–65 km distance classes, indicating populations spaced at these distances tended to be more dissimilar. The remaining distance classes were not significant.

## Discussion

Evaluating invasion pathways can be valuable for determining the properties of a successful species invasion and also can provide key information for identifying management priorities. Our data indicated that there has been a minimum of four independent introductions of bullfrogs into Montana from multiple diverse geographic sources, with little to no subsequent spread at the regional scale as implied by highly significant divergence between all populations. We also found low genetic diversity within invasive populations. These data together with evidence from previous studies on bullfrog invasions (Ficetola et al. 2008; Funk et al. 2011; Bai et al. 2012) suggest successful bullfrog establishment may not be limited by genetic variation. Furthermore, at a local scale within the YR, genetic haplotype data supported few introduction vectors and significant isolation by distance, a finding that is consistent with previous evidence for downstream dispersal in this river system (Sepulveda et al. 2015).

### Introduction history and genetic diversity of bullfrog invasions

Invasion events often involve demographic bottlenecks that are expected to result in low genetic variation in the colonizing population (e.g., Ficetola et al. 2008). Under this scenario, a “genetic paradox” emerges on how invading populations are capable of persisting despite low levels of variation available for adaptation (Sakai et al. 2001; Allendorf and Lundquist 2003). One solution is that multiple introductions from diverse sources bring together unusually large amounts of genetic variation and novel genetic combinations (Roman and Darling 2007; Simberloff 2009), allowing for persistence and high adaptive potential (e.g., Novak and Mack 1993; Kolbe et al. 2004; Lavergne and Molofsky 2007). For example, Kolbe et al. (2004) discovered that repeated brown anole lizard invasions from geographically disparate native sources resulted in elevated levels of genetic diversity in the introduced range. Beyond the number of introduction events, other factors affecting the genetic diversity of introduced populations include the diversity of the source, number of viable propagules, and extent of the bottleneck (Roman and Darling 2007). Regardless, some invasions have been successful even with low levels of genetic diversity (e.g., brown tree snakes in Guam; Richmond et al. 2015). Invasive populations of European bullfrogs remained drastically reduced in diversity relative to the native range due to a low number of initial founders and lack of subsequent mixing, despite at least six introductions (Ficetola et al. 2008).

Our study revealed multiple introductions and low genetic diversity in the introduced range (three of five populations had only one haplotype). Interestingly, populations with the highest genetic diversity (2–3 haplotypes; Yellowstone and Bitterroot) are widespread and abundant (Corn and Hendricks 1998; Sepulveda et al. 2015). Diversity was significantly lower than populations in the overlap native region where identical haplotypes were observed, but quantitatively similar to populations in the primary identified source region (i.e., west; Table 3). Funk et al. (2011) similarly observed no significant differences between native versus exotic bullfrog populations in Oregon. The multiple instances of established bullfrog populations with low variation in this and other systems (Ficetola et al. 2008; Funk et al. 2011; Bai et al. 2012) suggest that genetic diversity may not be a necessary condition for bullfrog colonization success. Furthermore, these data support previous assertions that the lack of genetic connectivity among introduced populations may not impede biological invasions (Dlugosch and Parker 2008).

### Vectors of bullfrog introduction and secondary spread

Progress in predicting propagule arrival and pressure begins with identifying the vectors responsible for movement of individuals among sites (Vander Zander and Olden 2008). While commercial bullfrog farming was likely responsible for historical introductions in Montana, the pet and bait trades are hypothesized to be the primary vectors for recent invasions in the YR, where bullfrogs were first recorded in public access sites popular for fishing and recreational uses (Sepulveda et al. 2015). Both trades are known to sell exotic species and have played a significant role in biological invasions (e.g., Strecker et al. 2011). For example, the bait trade and consequent disposal of worms and amphibians by anglers have been found to constitute a major vector for earthworm and salamander introductions (Riley et al. 2003; Keller et al. 2007). The use of baitfish is also common across North America (Drake and Mandrak 2014; Drake et al. 2015) and is still allowed in many waters of Montana, including the Yellowstone where bullfrogs occur. These trades are expected to continue posing a substantial risk for future invasions, particularly for herpetofauna (Kraus 2009). Therefore, slowing the spread of nonnatives will require legislation and efforts to both change the species sold at pet and bait stores and to change angler behavior. In 2005, the state of Montana designated bullfrogs as a prohibited species, which makes it illegal to possess, sale, purchase, exchange, or transport bullfrogs in Montana. Since this designation, no new populations have been detected, suggesting that legislative actions have thus far been effective for preventing new bullfrog introductions into Montana.

Another possible vector for bullfrog introductions is the aquaculture industry (Funk et al. 2011; Bai et al. 2012). Because of its large size, the bullfrog is a common frog species for farming. In China, the majority of feral bullfrog populations descended from individuals that escaped from nearby bullfrog farms (Liu and Li 2009). In Montana, the earliest documented introductions of bullfrogs are believed to have occurred as early as 1920 in the Bitterroot Valley (Black 1969). Historically, bullfrog farms operated both in the Bitterroot and near the Flathead (Mission Valley). Commercial frog farming is now uncommon in North America and, thus, is considered a low‐risk source for future bullfrog invasions into the region (Helfrich et al. 2009).

Preventing or slowing the secondary spread of invasive species to uninvaded sites has become an important management goal because impacts of invasions can be irreversible (Vander Zander and Olden 2008). Once introduced into a region, an invasive species can spread to habitats via natural or anthropogenic pathways. Many potential harmful species have established populations outside of their native range, yet may be far from achieving their potential geographic distribution (Vander Zander and Olden 2008). At a local scale in the YR, our genetic evidence and previous field data (Sepulveda et al. 2015) suggest that most secondary spread has occurred via natural pathways. Bullfrogs have been documented to occur in over 50 sites spanning 106 km, and breeding site occupancy increased nearly fourfold from 2010 to 2013 (Sepulveda et al. 2015). Here, we found evidence of genetic isolation by distance with a significant association between genetic and geographic (river) distance (Fig. 5). The proportion of one haplotype (MT1), in particular, decreased with increasing distance from the furthest identified upstream site (Fig. 4). While this does not reveal the directionality of gene flow, it does suggest that secondary spread of bullfrogs has occurred via natural pathways following introduction rather than through human‐mediated jumps (Wilson et al. 1999). This is also consistent with demographic data that suggest bullfrogs are dispersing more rapidly in a down‐ versus upstream direction (Sepulveda et al. 2015).

Evidence for the lack of gene flow among established bullfrog populations in our study region suggests that, at present, human‐mediated translocations and overland dispersal have not yet occurred over larger regional scales. This finding is in contrast to dispersal characteristics of invasive bullfrogs in the Colorado Front Range, which have been shown to be more likely to disperse over the landscape using overland routes rather than waterway corridors (Peterson et al. 2013). Also, translocations have been deemed responsible for large‐scale bullfrog invasions across Europe (Ficetola et al. 2007a) and in Mexico (Luja and Rodríguez‐Estrella 2010). In Montana, while natural movement is highly improbable given the large distances between sites (~100–700 km), we note that it is not possible to fully discount the role of human‐mediated movement within the state. For example, two haplotypes sampled in the Bitterroot (MT3) and Tongue (MT6) rivers are closely related (Fig. 2) and could be connected by translocation; under this scenario, small founding size followed by subsequent genetic drift could alternatively explain the high differentiation observed between them (Table 2). An understanding of the full complement of colonization pathways will be critical for improving policy actions, guiding integrative management strategies, and enhancing educational campaigns aimed at reducing the threat of future biological invasions.

### Comparisons with bullfrog invasions around the globe

The history of bullfrog initial introductions across the world is closely linked to intentional or incidental releases from commercial farms (Ficetola et al. 2007a), but the original sources of these bullfrogs and vectors of secondary spread vary among regions. At least three Montana bullfrog populations originated primarily from the western and overlap native groups, whereas European bullfrogs originated primarily from the western and eastern native groups (Ficetola et al. 2008), Willamette Valley bullfrogs came from the overlap zone (Funk et al. 2011), and Chinese bullfrogs originated from an introduced population in Cuba (Bai et al. 2012). Bullfrogs have been introduced to Montana a minimum of four times, whereas they have been introduced to Europe at least six times, to the Willamette Valley at least one time, and to China at least two times (Ficetola et al. 2008; Funk et al. 2011; Bai et al. 2012). It is surprising that the number of introductions in Montana is greater than that in China and similar to that in Europe given that China and Europe are orders of magnitude larger and more populated than Montana. For example, Europe is 26 times larger and has 725 times as many people. Given that Montana has a high number of minimum introductions relative to its area and population, a logical explanation is that residents were especially intent (e.g., high propagule pressure) on introducing bullfrogs for food or pets. If this was true, we would expect evidence of human‐aided translocations among Montana waters like in European and Mexican bullfrog populations (Ficetola et al. 2007a; Luja and Rodríguez‐Estrella 2010). Rather, we found the opposite; population differentiation was high and significant for all Montana population pairs indicating that humans have not translocated bullfrogs to other waters. This juxtaposition between the relatively high number of introductions and no apparent translocation suggests that Montana waters may have high habitat suitability, facilitating the establishment of introduced bullfrogs.

### Analytical considerations

This study highlights the utility of genetic approaches for providing valuable insights into the invasion dynamics of one of the most successful invaders in the world. Indirect genetic distance‐based methods are useful for making preliminary inferences, particularly regarding the origins, pathways, and genetic composition of invasions (Estoup and Guillemaud 2010). However, there has been considerable debate over the appropriate choice of marker given the timescale of interest (Wang 2010, 2011; Bohonak and Vandergast 2011), and we recognize that our use of a single mtDNA locus is limited in illuminating fine‐scale patterns of dispersal and contemporary gene flow (Wang 2010). It is also important to note that the loss in genetic diversity experienced during colonization bottlenecks is expected to be greater at mitochondrial than at nuclear genes, due to its lower effective population size (Birky‐Jr et al. 1989). Finally, the assumptions of equilibrium inherent in differentiation analyses are often violated in invading populations given an insufficient time since introduction and, thus, absolute Φ_ST_ estimates may be subject to bias (Fitzpatrick et al. 2012). Nonetheless, these data provided a glimpse into the past events leading to the current distribution of bullfrogs in Montana and further enabled comparisons with other introduced populations around the world. In the future, the use of additional genetic markers, including those representing adaptive variation (see Dlugosch et al. 2015), complemented by demographic data will be an imperative next step toward better understanding bullfrog invasion processes.

## Conclusions

Identifying natural and human‐mediated vectors of introduced species is important for preventing or slowing their spread and minimizing their ecological and economic impacts. For many species, however, intuition and anecdote have served, in the absence of any formal risk assessment, as the only basis for policy decisions and educational efforts. Quantitative approaches to dispersal and geographic spread can help to develop a more rigorous science on which to base these decisions. Once vectors are identified, managers can implement effective strategies that prevent or slow the spread of introduced species and minimize their impact.

Our findings of a lack of gene flow among introduced bullfrog populations emphasize the need to prioritize management actions aimed at stopping the importation of bullfrogs from outside Montana and on continuing education efforts to not transport bullfrogs within Montana. Fortunately, laws and regulations that ban the importation of bullfrogs and other invasive species into Montana are already in place, and our results suggest that continued support and enforcement of these laws and regulations should help prevent further introductions. These results also highlight the importance of genetic investigations for revealing key properties of biological species invasions that can inform wildlife conservation and management strategies.

## Conflict of Interest

None declared.

## Data Accessibility

Sequence data are available through GenBank, accession nos. KX344485–KX344492. Sample locations, R scripts, and Arlequin input files can be accessed from the Dryad Digital Repository: http://dx.doi.org/10.5061/dryad.g81c8.

## Supporting information


**Table S1.** *Lithobates* spp. mitochondrial cytochrome *b* haplotypes used in this study.
**Table S2.** Bullfrog haplotype composition by site along the Yellowstone River.
**Table S3.** Summary of invasive *Lithobates catesbeianus* sampling and 923‐bp mitochondrial cytochrome *b* haplotypes by drainage.
**Figure S1.** *Lithobates catesbeianus* native range sources of haplotypes detected in Montana and Wyoming.
**Figure S2.** Estimated smoothing curve for the generalized additive model of genetic distance (pairwise *F*_ST_) as a function of geographic distance.
