# Supplementary material for: Genetic reconstruction of a bullfrog invasion to elucidate vectors of introduction and secondary spread
Source: Ecol Evol. 2016 Jun 28;6(15):5221–33. doi: 10.1002/ece3.2278 (PMC4984499; doi:10.1002/ece3.2278)
Supplement: Supplementary file 1 — Table S1. Lithobates spp. mitochondrial cytochrome b haplotypes used in this study. Table S2. Bullfrog haplotype composition by site along the Yellowstone River. Table S3. Summary of invasive Lithobates catesbeianus sampling and 923‐bp mitochondrial cytochrome b haplotypes by drainage. Figure S1. Lithobates catesbeianus native range sources of haplotypes detected in Montana and Wyoming. Figure S2. Estimated smoothing curve for the generalized additive model of genetic distance (pairwise F ST) as a function of geographic distance. [file ECE3-6-5221-s001.pdf]

Supporting Information

Table S1. *Lithobates spp.* mitochondrial cytochrome *b* haplotypes used in this study.

GenBank No.	Species	Haplotype Name	Length (bp)	408bp Haplotype[1]	Citation
AY083288	*L. catesbeiana*	Rcat1319	924	H8	Austin *et al.* (2003)
AY083291	*L. catesbeiana*	Rcat1365	924	H17	Austin *et al.* (2003)
AY083292	*L. catesbeiana*	Rcat627	924	H7	Austin *et al.* (2003)
AY083293	*L. catesbeiana*	Rcat978	924	H1	Austin *et al.* (2003)
AY083294	*L. catesbeiana*	Rcat624	924	H7	Austin *et al.* (2003)
AY083295	*L. catesbeiana*	RcatM95	924	H9	Austin *et al.* (2003)
AY210362	*L. catesbeiana*	Rcat1245	925	H23	Austin *et al.* (2004)
AY210363	*L. catesbeiana*	Rcat1304	925	H14	Austin *et al.* (2004)
AY210364	*L. catesbeiana*	Rcat1415	925	H15	Austin *et al.* (2004)
AY210365	*L. catesbeiana*	Rcat1217	925	H11	Austin *et al.* (2004)
AY210366	*L. catesbeiana*	Rcat1340	925	H24	Austin *et al.* (2004)
AY210367	*L. catesbeiana*	Rcat650	925	H13	Austin *et al.* (2004)
AY210368	*L. catesbeiana*	RcatBF77	925	H25	Austin *et al.* (2004)
AY210369	*L. catesbeiana*	Rcat1228	925	H4	Austin *et al.* (2004)
AY210370	*L. catesbeiana*	Rcat1230	925	H2	Austin *et al.* (2004)
AY210371	*L. catesbeiana*	Rcat1293	925	H3	Austin *et al.* (2004)
AY210372	*L. catesbeiana*	Rcat1276	925	H6	Austin *et al.* (2004)
AY210373	*L. catesbeiana*	Rcat1088	925	H40	Austin *et al.* (2004)
AY210374	*L. catesbeiana*	Rcat1125	925	H39	Austin *et al.* (2004)
AY210375	*L. catesbeiana*	Rcat1071	925	H9	Austin *et al.* (2004)
AY210376	*L. catesbeiana*	Rcat1038	925	H16	Austin *et al.* (2004)
AY210377	*L. catesbeiana*	Rcat1112	925	H9	Austin *et al.* (2004)
AY210378	*L. catesbeiana*	Rcat1222	925	H10	Austin *et al.* (2004)
AY210379	*L. catesbeiana*	Rcat5	408	H5	Austin *et al.* (2004)
AY210380	*L. catesbeiana*	Rcat12	408	H12	Austin *et al.* (2004)
AY210381	*L. catesbeiana*	Rcat18	408	H18	Austin *et al.* (2004)
AY210382	*L. catesbeiana*	Rcat19	408	H19	Austin *et al.* (2004)
AY210383	*L. catesbeiana*	Rcat20	408	H20	Austin *et al.* (2004)
AY210384	*L. catesbeiana*	Rcat21	408	H21	Austin *et al.* (2004)
AY210385	*L. catesbeiana*	Rcat22	408	H22	Austin *et al.* (2004)
AY210386	*L. catesbeiana*	Rcat26	408	H26	Austin *et al.* (2004)
AY210387	*L. catesbeiana*	Rcat27	408	H27	Austin *et al.* (2004)
AY210388	*L. catesbeiana*	Rcat28	408	H28	Austin *et al.* (2004)
AY210389	*L. catesbeiana*	Rcat29	408	H29	Austin *et al.* (2004)
AY210390	*L. catesbeiana*	Rcat30	408	H30	Austin *et al.* (2004)
AY210391	*L. catesbeiana*	Rcat31	408	H31	Austin *et al.* (2004)
AY210392	*L. catesbeiana*	Rcat32	408	H32	Austin *et al.* (2004)
AY210393	*L. catesbeiana*	Rcat33	408	H33	Austin *et al.* (2004)
AY210394	*L. catesbeiana*	Rcat34	408	H34	Austin *et al.* (2004)
AY210395	*L. catesbeiana*	Rcat35	408	H35	Austin *et al.* (2004)
AY210396	*L. catesbeiana*	Rcat36	408	H36	Austin *et al.* (2004)

GenBank No.	Species	Haplotype Name	Length (bp)	408bp Haplotype[1]	Citation
AY210397	*L. catesbeiana*	Rcat37	408	H37	Austin *et al.* (2004)
AY210398	*L. catesbeiana*	Rcat38	408	H38	Austin *et al.* (2004)
AY210399	*L. catesbeiana*	Rcat41	408	H41	Austin *et al.* (2004)
KX344485	*L. catesbeiana*	Lcat-MT1	937	H1	this study
KX344486	*L. catesbeiana*	Lcat-MT2	937	H5	this study
KX344487	*L. catesbeiana*	Lcat-MT3	937	H2	this study
KX344488	*L. catesbeiana*	Lcat-MT4	937	H9	this study
KX344489	*L. catesbeiana*	Lcat-MT5	937	H11	this study
KX344490	*L. catesbeiana*	Lcat-MT6	937	H2	this study
KX344491	*L. catesbeiana*	Lcat-MT7	937	H11	this study
KX344492	*L. catesbeiana*	Lcat-WY8	937	H7	this study
AY083281	*L. clamitans*	Rcla986	924	--	Austin *et al.* (2003)
AY083299	*L. heckscheri*	Rhec1407	924	--	Austin *et al.* (2003)
AY083286	*L. okaloosae*	Roka1404	924	--	Austin *et al.* (2003)
AY083273	*L.septentrionalis*	Rsep24410	924	--	Austin *et al.* (2003)

[1]408 bp cytochrome b haplotypes from Austin et al. (2004); Some longer length (924-937 bp) sequences share a haplotype due to 100% identity along the 408 bp region.

Table S2. Bullfrog cyt *b* haplotype composition by site along the Yellowstone River. For each site, sample size *(n*), proportion of each haplotype (MT1, MT2), and river distance (km) from most upstream site (3_11) are reported.

Site	Distance from 3_11 (km)	*N*	MT1	MT2
3_11	0.00	28	1.000	0.000
Audubob channel	9.74	9	0.556	0.444
4_2	11.65	12	0.000	1.000
1_1	14.01	2	1.000	0.000
KOA	14.76	14	0.571	0.429
3_4	21.36	24	0.875	0.125
5_13	25.03	5	1.000	0.000
Weaver bridge	26.21	24	0.167	0.833
7_9	26.82	12	0.833	0.167
Barb's pond	27.05	14	1.000	0.000
7_14	27.37	13	0.231	0.769
6_5	32.03	2	0.000	1.000
7_3	35.15	12	0.417	0.583
7_11	36.56	30	0.533	0.467
6_2	38.49	2	0.000	1.000
7_7	40.02	13	0.923	0.077
RACA_263	40.57	7	0.714	0.286
7_16	42.01	14	0.214	0.786
5_16	45.24	7	0.857	0.143
RACA_275	48.18	5	0.200	0.800
5_2b	52.11	1	0.000	1.000
YMA	57.67	4	0.000	1.000
7_10b	57.82	1	1.000	0.000
RACA_284	58.96	9	0.556	0.444
7_1	60.23	1	0.000	1.000
RACA_310b	62.90	3	0.333	0.667
RACA_310	62.91	2	0.000	1.000
RACA_309	63.13	4	0.500	0.500
RACA_309b	63.19	3	0.333	0.667
7_8	64.22	17	0.235	0.765
8_18	68.51	3	0.000	1.000
8_16	74.11	10	0.000	1.000
8_27	100.11	1	1.000	0.000

Table S3. Summary of invasive *Lithobates catesbeianus* sampling and mitochondrial cyt *b* haplotypes by drainage.

Location	State	Lat	Long	*N*	MT1	MT2*	MT3*	MT4	MT5*	MT6	MT7	WY8
Yellowstone River (YR)	MT	45.782	-108.501	308	163	145	0	0	0	0	0	0
Bitterroot River (BR)	MT	46.523	-114.105	40	0	0	18	8	0	0	14	0
Flathead River (FR)	MT	47.393	-114.569	5	0	0	0	0	5	0	0	0
Tongue River (TR)	MT	45.339	-106.318	15	0	0	0	0	0	15	0	0
Grand Teton NP (GTNP)	WY	43.639	-110.616	27	0	0	0	0	0	0	0	27

Location abbreviations in parentheses

** Montana 923 bp haplotypes identical to those reported in Austin et al. (2004): MT2 = H5, MT3 = H2, MT5 = H11*

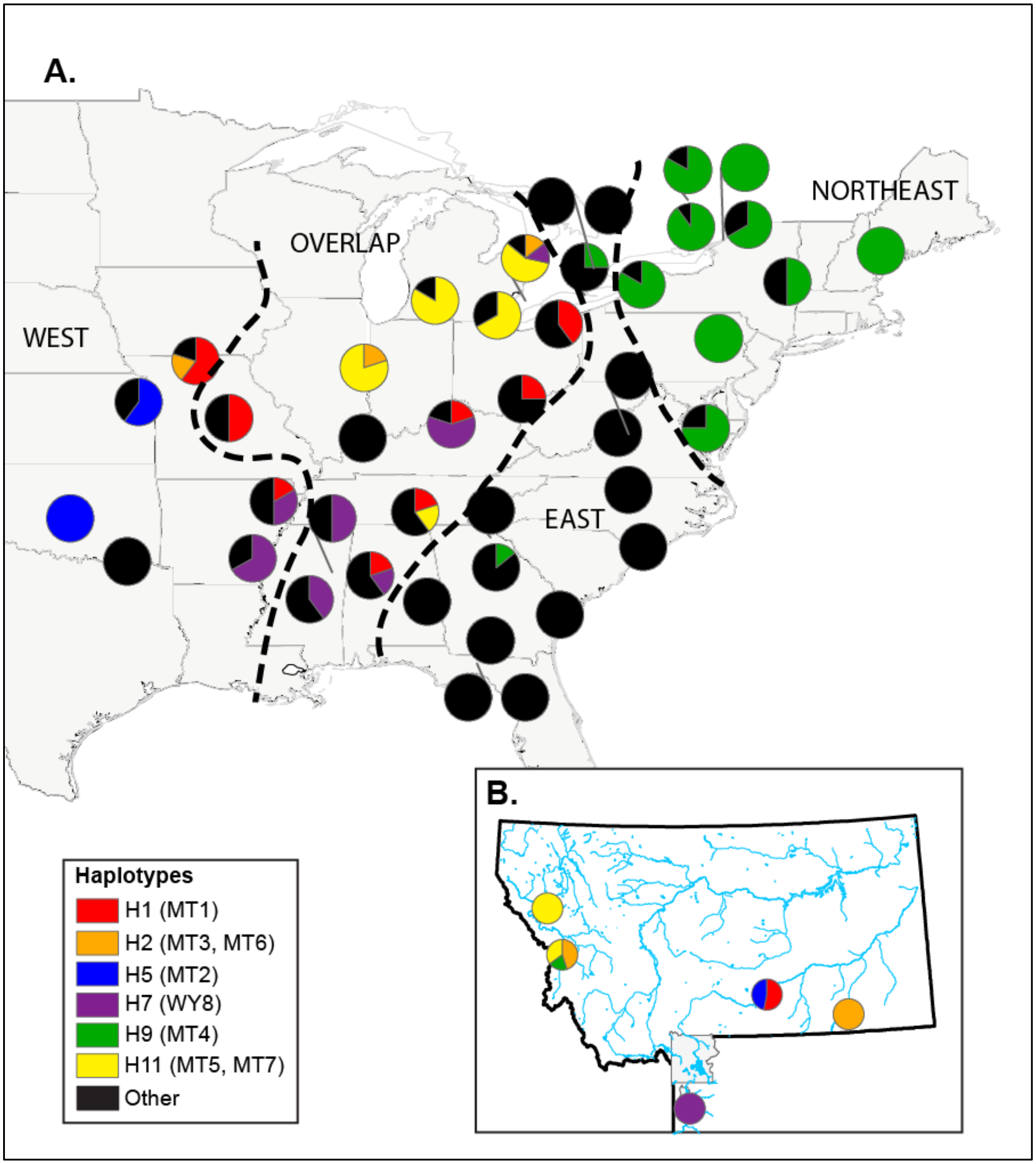

Figure S1. *Lithobates catesbeianus* native range sources (A) of cyt *b* haplotypes detected in Montana and Wyoming (B). Haplotype distribution when data was limited to 408 bp of the cyt *b* locus. Native range regions (west, overlap, east, northeast) were defined by nested clade analysis as previously reported in Austin *et al.* (2004). Montana and Wyoming haplotypes that are identical at the 408 bp region are indicated in parentheses.

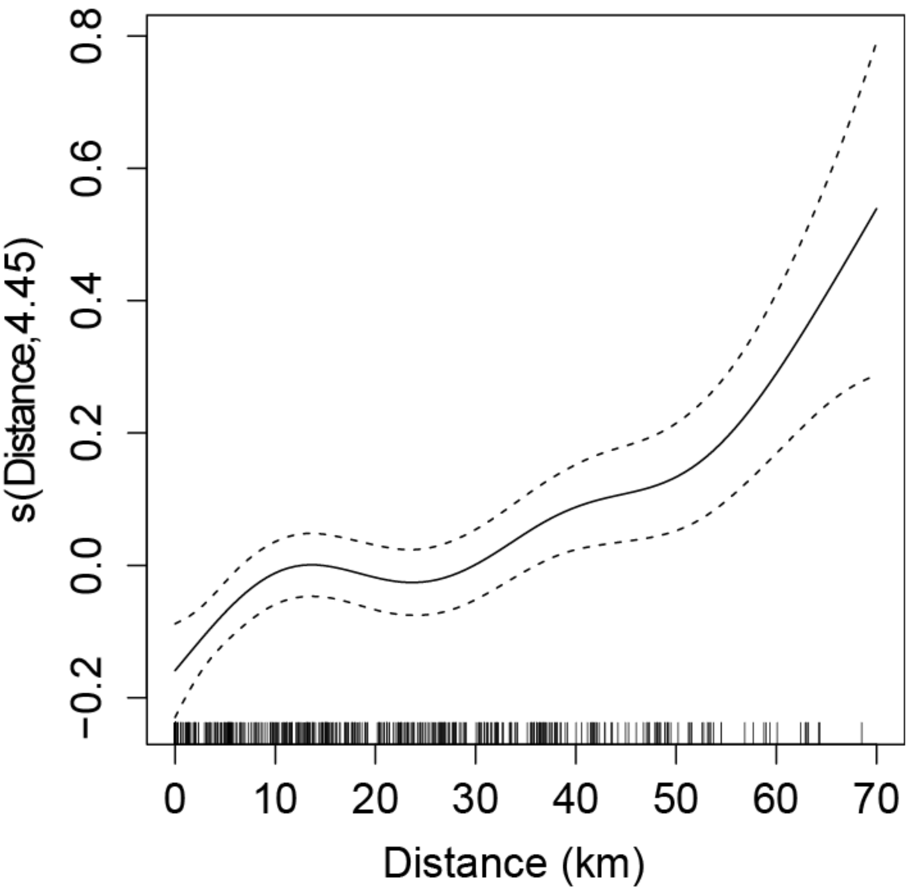

Figure S2. Estimated smoothing curve for the generalized additive model of genetic distance (pairwise F_{ST}) as a function of geographic distance in kilometers (km). The solid line represents the smoother and the dotted lines are 95% point-wise confidence bands on the predicted values. Significance of the smoothing curve = 3.5e-9.

Literature Cited

Austin JD, Lougheed SC, Boag PT (2004) Discordant temporal and geographic patterns in maternal lineages of eastern north American frogs, *Rana catesbeiana* (Ranidae) and *Pseudacris crucifer* (Hylidae). *Molecular Phylogenetics and Evolution* **32**, 799-816.

Austin JD, Lougheed SC, Moler PE, Boag PT (2003) Phylogenetics, zoogeography, and the role of dispersal and vicariance in the evolution of the *Rana catesbeiana* (Anura : Ranidae) species group. *Biological Journal of the Linnean Society* **80**, 601-624.
